# Launay’s External Carotid Vein

**DOI:** 10.3390/medicina57090985

**Published:** 2021-09-18

**Authors:** Mihaela Daniela Manta, Adelina Maria Jianu, Mugurel Constantin Rusu, Şerban Arghir Popescu

**Affiliations:** 1 Department of Anatomy, Faculty of Medicine, Victor Babeș University of Medicine and Pharmacy, 300041 Timișoara, Romania; monea.mihaela@umft.ro; 2 Faculty of Dental Medicine, Carol Davila University of Medicine and Pharmacy, 050474 Bucharest, Romania; 3 Faculty of Medicine, Carol Davila University of Medicine and Pharmacy, 050474 Bucharest, Romania; serban.popescu@umfcd.ro

**Keywords:** carotid artery, jugular vein, parotid, retromandibular, mandible, occipitoauricular trunk, extracondylar vein, deep temporal veins

## Abstract

*Background and Objectives*: Launay’s external carotid vein (ECV) is poorly represented in the anatomical literature, although it is an occasional satellite of the external carotid artery (ECA). We aimed to establish the incidence and morphology of the ECV. *Materials and Methods*: One hundred computed tomography angiograms were investigated, and ECVs were documented anatomically, when found. *Results*: Launay’s vein was found in 3/200 sides (1.5%) in a male and two female cases. In two of these cases, the ECV was a replaced variant of the anterior division of the retromandibular vein (RMV), and the facial vein (FV) ended in the external jugular vein. In the third case with the ECV, the RMV was absent and the common FV that resulted from that ECV and the FV drained into the internal jugular vein. The ECV could also appear as an accessory RMV, not just as a replaced one. Additional variants were found, such as fenestration of the external jugular vein (EJV), the extracondylar vein draining the deep temporal veins and an arterial occipitoauricular trunk. *Conclusions*: Surgical dissections of the ECA in the retromandibular space should carefully observe an ECV to avoid unwanted haemorrhagic events. Approaches of the neck of the mandible should also carefully distinguish the consistent extracondylar veins.

## 1. Introduction

Commonly, the retromandibular vein (RMV) forms within the parotid space from the superficial temporal and maxillary veins [1]. The posterior division of the RMV and the posterior auricular vein further form the external jugular vein (EJV) which drains into the subclavian vein [2]. The anterior division of the RMV joins the facial vein (FV) proper (or anterior FV), to form the common FV which, in turn, empties into the internal jugular vein (IJV). The RMV is the most used and validated anatomical structure to predict the intraparotid course of the facial nerve [3]. Deep to the RMV courses the external carotid artery (ECA) that, commonly, has no satellite vein. This is because the branches of the facial nerve are just lateral to the RMV [4].

An important number of studies have previously described the numerous possibilities of anatomic variations of the veins of the neck. Such variations were documented by Henle (1868) [5], as well as in Bergman’s Encyclopedia of Human Anatomic Variation [6]. However, the variant external carotid vein (ECV) of Launay (1868–1962) [7,8] was not found by most researchers, or it was overlooked when the veins of the head and neck were documented. The ECV was documented by Caroline Mage in her “Thèse pour l’obtention du Diplome d’Etat de Docteur en Medecine Qualification en Chirurgie Générale” [9]. This author observed the textbook of Rouvière and Delmas (1985) where the ECV is drawn in figures 168–170 [10]. As documented by Mage (2016), the ECV is a satellite of the external carotid artery (ECA) and could either replace or double the RMV [11,12]. As drawn in Paturet’s textbook, the ECV unites an upper parotid venous confluent receiving the superficial temporal and maxillary veins with a lower hyoid venous confluent joined by the facial and lingual veins [9,12]. As drawn in Paturet, the ECV could also connect directly to the IJV [9,12]. Henle (1868) presented a drawing in which the superficial temporal vein can be observed dividing inferiorly in two branches: (a) a posterior one, draining into the EJV and (b) an anterior one, larger, indicated as “vena facialis posterior”, coursing deep to the stylohyoid muscle (as the ECA also does) to join the anterior FV and form the common FV [5].

The present study was therefore aimed at documenting on computed tomography (CT) angiograms the anatomy of the ECV.

## 2. Materials and Methods

For the investigation of the ECV, randomly documented and archived CT angiograms of 100 patients, 48 males and 52 females, were used. They were scanned to elucidate whether or not vascular variants or pathology could justify their cerebral insufficiency symptoms. For the CT studies, an iodine radiocontrast agent was injected into the brachial vein, followed by a saline medium. CT was performed with a 32-slice scanner (Siemens Multislice Perspective Scanner) using a 0.6 mm collimation and a reconstruction of 0.75 mm thickness with 50% overlap for the multiplanar, maximum intensity projection and 3D volume rendering technique [13]. The arterial variants were documented with the Horos for iOS (Horos Project) software, as shown in previous studies [14].

## 3. Results

In three cases (3%, one male and two female patients), the unilateral presence of the ECV was documented, and no pathological vascular changes were detected in these patients. There were, however, peculiar differences between these three cases, all of which were positive for Launay’s vein. The differences will be further presented here case by case.

### 3.1. The First Variant of the ECV

**Figure 1:**
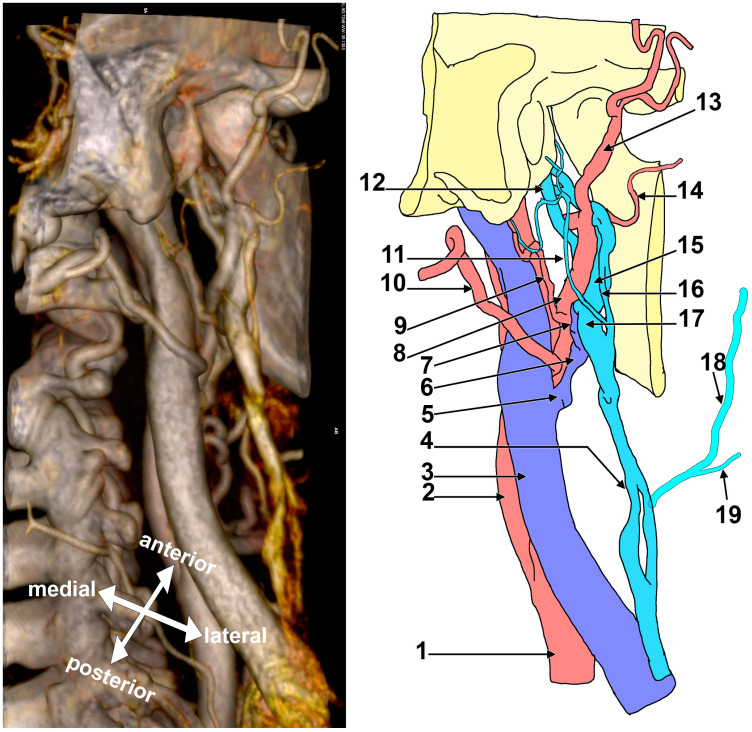
Right postero-infero-lateral view of the retromandibular fossa. Three-dimensional volume rendering and drawing (the branches of the external carotid artery in the carotid triangle were omitted). 1. Common carotid artery; 2. internal carotid artery; 3. internal jugular vein; 4. fenestrated external jugular vein; 5. thyro-linguo-facial trunk; 6. external carotid vein (Launay’s vein); 7. antero-medial branch of the retromandibular vein; 8. external carotid artery; 9. posterior auricular artery; 10.occipital artery; 11. posterior auricular vein; 12. posterior pharyngeal trunk; 13. superficial temporal artery; 14. transverse facial artery; 15. deep limb of the fenestrated retromandibular vein; 16. superficial limb of the fenestrated retromandibular vein; 17. postero-lateral branch of the retromandibular vein. Also included are the facial vein (18), which drains in the anterior arm of the fenestrated external jugular vein, and a submental tributary (19) of the facial vein.

In Case #1 (Figure 1), a fenestrated RMV was first identified with two unequal limbs, a thin superficial limb and a large deep limb. The limbs of the RMV were supplied by a complicated venous network built up by posterior pharyngeal veins and the superficial temporal, maxillary, posterior auricular and occipital veins. The terminal segment of the ECA and its branches were intermingled with that venous network. The two limbs of the RMV further joined to continue as the EJV on the sternocleidomastoid muscle. On that muscle, the EJV was fenestrated, with the anterior limb of that fenestration joined by the FV. From the posterior limb of the RMV branched the ECV. That ECV continued on the anterior side of the ECA and drained into the IJV.

### 3.2. The Second Variant of the ECV

**Figure 2:**
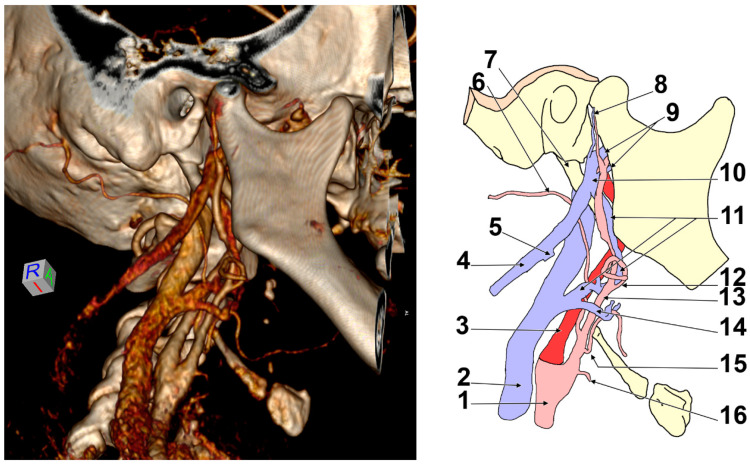
Right infero-lateral view of the retromandibular fossa and carotid triangle. Three-dimensional volume rendering and drawing. 1. Common carotid artery; 2. internal jugular vein; 3. internal carotid artery; 4. external jugular vein; 5. terminal end of the facial vein; 6. occipital artery; 7. styloid process; 8. superficial temporal artery and vein; 9. maxillary veins; 10. retromandibular vein; 11. Launay’s external carotid vein; 12. facial artery; 13. external carotid artery; 14. thyro-lingual venous trunk; 15. lingual artery; 16. superior thyroid artery.

In Case #2 (Figure 2), on the right side of the head were two veins coursing through Juvara’s buttonhole (medially to the base of the neck of the mandible), one larger vein above the maxillary artery and another thinner vein, located inferior to the maxillary artery. These were considered maxillary veins. The thinner inferior maxillary vein coursed on the infraincisural triangle of the mandible, being supplied by the masseteric vein and the pterygoid plexus. The superior maxillary vein was consistently supplied by the pterygoid plexus.

Posteroinferior to the mandible’s neck was the superior maxillary vein, which joined the superficial temporal vein to form a short RMV. This immediately divided into two dichotomous veins, posterior and anterior, after joining the inferior maxillary vein. The posterior vein was the EJV, which continued on to the sternocleidomastoid muscle and joined the FV. The anterior division of the RMV was Launay’s ECV, which coursed through the anterior side of the ECA toward the carotid triangle. In the carotid triangle, the ECV joined a thyrolingual venous trunk to eventually end into the IJV. The ECV and the thyrolingual trunk formed the venous forceps through which the ECA was coursing through.

### 3.3. The Third Variant of the ECV

**Figure 3:**
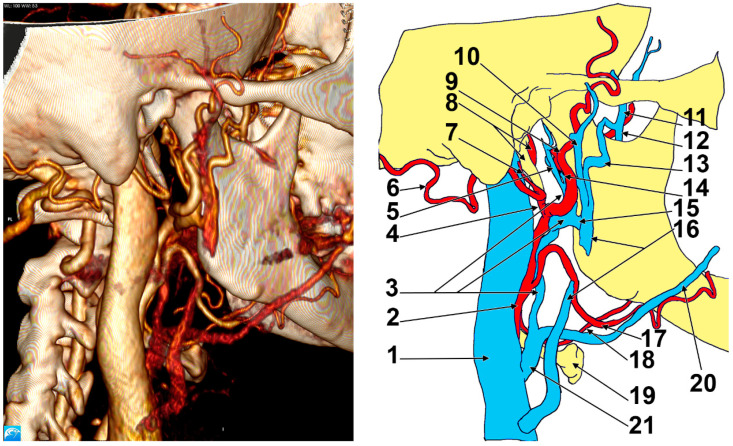
Right postero-infero-lateral view of the retromandibular fossa. Three-dimensional volume rendering and drawing. 1. Internal jugular vein; 2. external carotid artery; 3. Launay’s external carotid vein; 4. occipitoauricular trunk; 5. retrocondylar vein; 6. occipital artery; 7. posterior auricular artery; 8. internal carotid artery, styloid process; 9. superficial temporal artery; 10. superficial temporal vein; 11. deep temporal venous trunk; 12. communicating vein with the maxillary vein; 13. extracondylar vein; 14.maxillary vein; 15. intraparotid communicating vein; 16. external jugular vein (interrupted); 17. facial artery; 18. lingual artery; 19. hyoid bone; 20. proper facial vein; 21. common facial vein.

**Figure 4:**
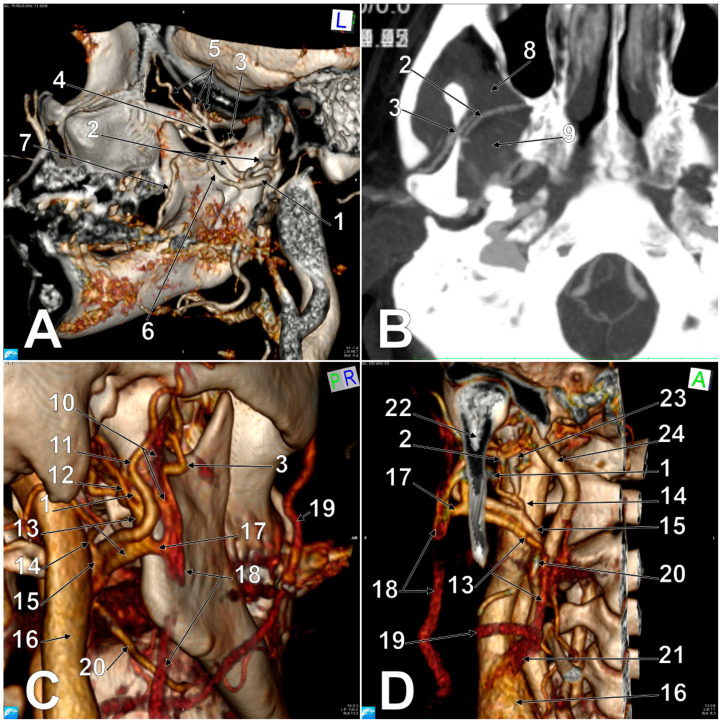
Details of the anatomic variations in Case #3. (**A**). Three-dimensional volume rendering, medial view of the right mandibular ramus. (**B**). Axial reconstructed slice through the right mandibular notch. (**C**). Three-dimensional volume rendering, postero-infero-lateral view of the right mandibular ramus. (**D**). Three-dimensional volume rendering, anterior view of Launay’s vein. 1. Maxillary vein; 2. maxillary artery; 3. extracondylar vein; 4. deep temporal venous trunk; 5. deep temporal veins; 6. communicating vein between the extracondylar and maxillary veins; 7. buccal vein; 8. temporal muscle; 9. lateral pterygoid muscle; 10. superficial temporal vein; 11. superficial temporal artery; 12. retrocondylar vein; 13. Launay’s external carotid vein; 14. occipitoauriaular trunk; 15. external carotid artery; 16. internal jugular vein; 17. anastomosis of the external carotid and external jugular veins; 18. external jugular vein; 19. proper facial vein; 20. facial artery; 21. common facial vein; 22. neck of mandible; 23. styloid process; 24. internal carotid artery.

In Case #3 (Figure 3 and Figure 4), on the right side of the head the EJV resulted from the superficial temporal and maxillary veins and was located posterior to the masseter muscle and posterolateral to the posterior border of the mandibular ramus. The superficial temporal vein was 1.87 cm long and resulted from a frontal and a parietal tributary, which joined together inferior to the posterior zygomatic root. Atypically, the intraparotid superficial temporal vein received, at 0.5 cm above its junction with the maxillary vein, a large extracondylar vein. That extracondylar vein was further studied, and it was found to have formed medially to the mandibular notch from a descending deep temporal venous trunk supplied by three deep temporal tributaries and a communicating branch with the maxillary vein, which coursed parallel and inferior to the maxillary artery and was applied on the infraincisural triangle of the mandible. That communicating branch, in turn, received a buccal vein coursing through the tendon of the temporalis muscle. The extracondylar vein coursed through the mandibular notch, continued deep to the posterior fascicle of the masseter muscle, then lateral to the base of the neck of the mandible and finally joined the superficial temporal vein.

At 0.39 cm before joining the superficial temporal vein, the maxillary vein sent off an inferior trunk that was joined by a retrocondylar vein to form Launay’s ECV. The initial segment of the ECV was anteromedial to the ECA’s terminal segment. Then, it anastomosed with the EJV and continued along the ECA and in front of it. In that course, the ECV crossed medially to the facial artery and joined the FV to form a common trunk (common FV) of 1.05 cm in length, which crossed anteriorly to the carotid bifurcation and ended in the IJV.

An additional anatomic variant was found on the right side: an occipitoauricular trunk left of the ECA that further divided into the occipital and posterior auricular arteries.

## 4. Discussion

**Figure 5:**
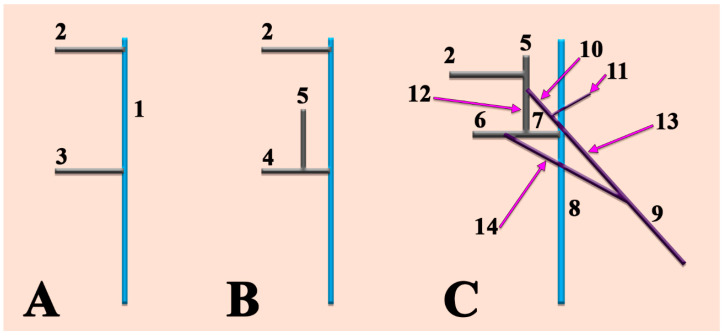
Schematic representation of successive embryonic stages of venous development. (**A**). 10 mm embryo; (**B**).18 mm embryo; (**C**). 40 mm embryo. 1. Precardinal vein; 2. primitive maxillary vein; 3. ventral pharyngeal vein; 4. linguofacial vein; 5. retromandibular vein; 6. proper facial vein; 7. common facial vein; 8. internal jugular vein; 9. external jugular vein; 10. posterior division of the retromandibular vein; 11. posterior auricular vein; 12. anterior division of the retromandibular vein; 13. posterior connection of the external jugular vein; 14. anterior connection of the external jugular vein.

Although each of these three cases with ECVs had a specific venous design, they had a common trait: the usual tributaries of the RMV (i.e., the superficial temporal and maxillary veins) equally contributed to the origin of the EJV. Moreover, the RMV, when formed (cases #1 and #2), contributed to the formation of Launay’s ECV. However, although variations of the formative tributaries of the EJV, such as the posterior division of the RMV, the posterior auricular vein or the maxillary vein, are occasionally found [15], Launay’s ECV has been scarcely represented in anatomic reports. According to the diagrams of Dorcas Hager Padget and the embryologic explanations of Choudhry et al. [15,16,17], the anterior division of the primitive RMV joins the FV to form the common FV, which, in turn, drains into the IJV (Figure 5). According to its final course, the division of the RMV becomes either the definitive RMV or an ECV coursing with the ECA. When the anterior division of the RMV is absent, the RMV drains completely into the EJV [18]. However, when the posterior division of the RMV is absent (undivided RMV), it leads to the absence of the EJV [19]. The persistence of the anterior connection of the ECV leads to the EJV termination of the FV, such as in Cases #1 and #2 reported here. This variant occurs rarely [15].

**Figure 6:**
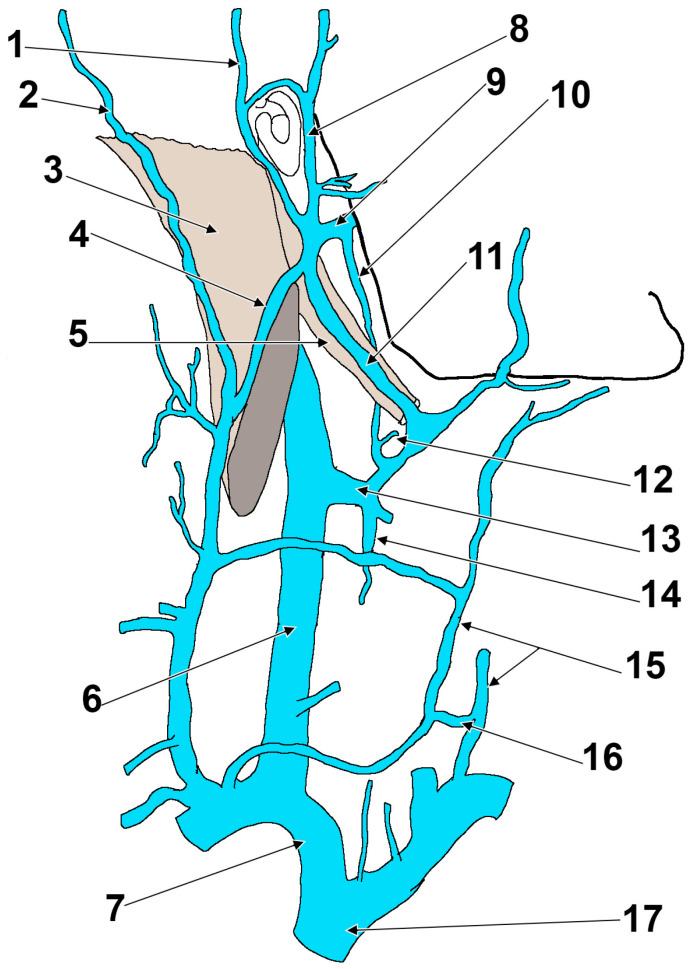
Drawing, modified after Rouvière and Delmas (1985), depicting the veins on the right side of the neck. 1. Posterior auricular vein; 2. superficial occipital vein; 3. sternocleidomastoid m.; 4. external jugular vein; 5. posterior belly of digastric m.; 6. internal jugular vein; 7. right brachiocephalic vein; 8. superficial temporal vein; 9. maxillary vein; 10. external carotid vein of Launay; 11. intraparotid communicating vein; 12. lingual vein; 13. thyro-linguo-facial trunk; 14. superior thyroid vein; 15. anterior jugular vein; 16. jugular venous arch; 17. superior vena cava.

In their well-known textbook of anatomy, Rouvière and Delmas presented a variant of the ECV (Figure 6), one that is different from all those we found and reported here: this variant showed the ECV origin from the maxillary vein, such as in Case #3 above, but the maxillary vein continued further towards a venous trunk that was sending off both the RMV and EJV. In the presentation by Rouvière and Delmas, the RMV and the FV formed the common FV, which joined the ECV before emptying into the IJV. Therefore, this variant presents the ECV as an ‘accessory RMV’ coursing deep into the stylohyoid muscle. The ECV we found appeared as a ‘replaced RMV’ with a deep course.

Different authors have reported on Launay’s vein, but without applying the correct term, ECV, and without assigning it the eponym. Gaughran (1961) described the entrance of the ECA in the parotid compartment and indicated that the artery has a ‘satellite vein’ or a ‘venous satellite’ [20]. Patil et al. (2014) found an ‘anonymous vein’ that ‘descended deep to the stylohyoid and posterior belly of the digastric muscle, crossed superficially the external carotid artery, and drained into the internal jugular vein’ [21]. The RMV was absent in that case. Thus, these authors found the ‘replaced RMV’ variant of Launay’s ECV.

In Case #3, a peculiar drainage of the deep temporal veins was found towards the EJV through the extracondylar vein and the communicating branch with the maxillary vein. This was rather unexpected, as the deep temporal veins commonly drain into the pterygoid plexus. Such consistent veins could be accidentally punctured during anaesthetic infiltrations, either within the mandibular notch or high in the pterygomandibular space.

Surgery is the only treatment for parotid gland tumours, and the surgical approaches of the parotid gland should avoid any facial nerve damage [3]. The RMV is the most used landmark to predict the course of the facial nerve within the parotid gland based on the assumption that the nerve courses lateral to that vein [3,4]. As the facial nerve branches terminally within the parotid gland on the lateral side of the intraparotid veins, a variant ECV would, seemingly, not alter the venous surgical landmark. However, as the ECV is applied to the ECA, care should be taken when these vessels are surgically dissected. In this regard, when ligation of the ECA is decided and performed, care should be taken if an ECV is applied to the ECA. Moreover, surgical approaches of the palatine tonsil should spare not only the internal and external carotid arteries and ECA branches [22], but also the Launay ECV, if present. Nevertheless, orthognathic surgeries, such as bilateral sagittal split osteotomy, reach close to the immediate retromandibular vascular elements, which should be carefully documented.

The mandibular notch is the ideal point at which anaesthetic can be delivered to achieve a masseteric nerve block [23]. Therefore, vascular variants, such as the extracondylar vein we found in Case #3, would add haemorrhagic risks to an anaesthetic puncture at the mandibular notch.

## 5. Conclusions

The ECV courses along the ECA above and below the stylohyoid muscle. Care should be taken when surgical dissections of the ECA are planned in order to avoid damaging the ECV and, in turn, haemorrhagic events. Surgeons should be aware that if a normal RMV does not contribute to the common FV formation, an ECV could do so and should be either spared or ligated. Retromandibular venous variations are a common finding; thus, parotid dissections should be carefully performed. A venous pathway through the mandibular notch could link the deep temporal veins to the veins of the parotid space. Therefore, the ECV should be better promoted during anatomical teaching. Nevertheless, this case series might not reflect the true prevalence of Launay’s vein in a real population. It is suggested that further studies still have to determine the prevalence of Launay’s vein.

## Data Availability

No new data were created or analyzed in this study. Data sharing is not applicable to this article.
